# Sustainable Innovation: Fabrication and Characterization of Mycelium-Based Green Composites for Modern Interior Materials Using Agro-Industrial Wastes and Different Species of Fungi

**DOI:** 10.3390/polym16040550

**Published:** 2024-02-18

**Authors:** Worawoot Aiduang, Kritsana Jatuwong, Praween Jinanukul, Nakarin Suwannarach, Jaturong Kumla, Wandee Thamjaree, Thana Teeraphantuvat, Tanut Waroonkun, Rawiwan Oranratmanee, Saisamorn Lumyong

**Affiliations:** 1 Office of Research Administration, Chiang Mai University, Chiang Mai 50200, Thailand; worawoot.aiduang@cmu.ac.th (W.A.); kritsana.ja@cmu.ac.th (K.J.); suwan.462@gmail.com (N.S.); jaturong_yai@hotmail.com (J.K.); 2 Department of Biology, Faculty of Science, Chiang Mai University, Chiang Mai 50200, Thailand; 3 Faculty of Architecture, Chiang Mai University, Chiang Mai 50200, Thailand; praween_ji@cmu.ac.th (P.J.); tanut.w@cmu.ac.th (T.W.); rawiwan.o@cmu.ac.th (R.O.); 4 Center of Excellence in Microbial Diversity and Sustainable Utilization, Chiang Mai University, Chiang Mai 50200, Thailand; 5 Department of Physics and Materials Science, Faculty of Science, Chiang Mai University, Chiang Mai 50200, Thailand; wandee.th@cmu.ac.th; 6 Northfield Mount Hermon School, Mount Hermon, MA 01354, USA; kennythana@gmail.com; 7 Academy of Science, The Royal Society of Thailand, Bangkok 10300, Thailand

**Keywords:** agro-industrial wastes, eco-sustainability, green materials, mycelium technology, SDGs8 and 9

## Abstract

Mycelium-based bio-composites (MBCs) represent a sustainable and innovative material with high potential for contemporary applications, particularly in the field of modern interior design. This research investigates the fabrication of MBCs for modern interior materials using agro-industrial wastes (bamboo sawdust and corn pericarp) and different fungal species. The study focuses on determining physical properties, including moisture content, shrinkage, density, water absorption, volumetric swelling, thermal degradation, and mechanical properties (bending, compression, impact, and tensile strength). The results indicate variations in moisture content and shrinkage based on fungal species and substrate types, with bamboo sawdust exhibiting lower shrinkage. The obtained density values range from 212.31 to 282.09 kg/m^3^, comparable to traditional materials, suggesting MBCs potential in diverse fields, especially as modern interior elements. Water absorption and volumetric swelling demonstrate the influence of substrate and fungal species, although they do not significantly impact the characteristics of interior decoration materials. Thermal degradation analysis aligns with established patterns, showcasing the suitability of MBCs for various applications. Scanning electron microscope observations reveal the morphological features of MBCs, emphasizing the role of fungal mycelia in binding substrate particles. Mechanical properties exhibit variations in bending, compression, impact, and tensile strength, with MBCs demonstrating compatibility with traditional materials used in interior elements. Those produced from *L. sajor-caju* and *G. fornicatum* show especially promising characteristics in this context. Particularly noteworthy are their superior compression and impact strength, surpassing values observed in certain synthetic foams multiple times. Moreover, this study reveals the biodegradability of MBCs, reaching standards for environmentally friendly materials. A comprehensive comparison with traditional materials further supports the potential of MBCs in sustainable material. Challenges in standardization, production scalability, and market adoption are identified, emphasizing the need for ongoing research, material engineering advancements, and biotechnological innovations. These efforts aim to enhance MBC properties, promoting sustainability in modern interior applications, while also facilitating their expansion into mass production within the innovative construction materials market.

## 1. Introduction

During the past two decades, polymer-based materials have been growing in popularity across a variety of worldwide industries, due to their flexibility, durability, and simplicity of use, which makes it impossible to think about any product that has not been created from this type of material. Most synthetic polymer materials are primarily derived from petroleum and coal as raw materials, which makes them environmentally unfriendly and causes several problems with the environment because they cannot be incorporated into a natural recycling system [1,2]. In an era of growing environmental awareness and the pressing need to address climate change, sustainable innovation has become recognized as an important part of modern science and technology [3]. One of the primary challenges that we currently face is the discovery and development of environmentally friendly materials for a variety of industries that can reduce the environmental impact associated with conventional materials, particularly within the building material sectors related to various interior elements [4,5,6].

Green composites are currently one of the modern environmentally friendly materials that have attracted a lot of interest in a variety of interior designs as well as other sectors due to their natural biodegradability, low environmental impact, and feasibility from a technological and economic standpoint [7,8]. Many kinds of green composites present a viable alternative for environmentally friendly materials, heralding a paradigm change across a range of industries where environmental responsibility and sustainability have been given top priority. A groundbreaking approach to sustainable materials, focusing on the utilization of agro-industrial wastes can serve as the basis for a portfolio of environmentally friendly and sustainable products in order to replace products derived from petroleum sources [8]. One of the modern materials that exhibits great potential as a green composite material among these groundbreaking findings are MBCs, which are generated through a 100% biotechnological process using a variety of fungal species combined with agro-industrial wastes [9,10]. This material has previously demonstrated great potential and enormous possibility as a renewable material used in various industries, such as construction, furniture, household goods, and agriculture, along with packaging, by offering a biodegradable and sustainable substitute for conventional synthetic materials and composites [11,12,13]. Importantly, the production process has been low-cost and generates a slight carbon footprint [13].

This research investigates the biosynthesis and fabrication of MBCs, utilizing a variety of fungal species in combination with agro-industrial waste materials generated during processing activities. These materials, including bamboo sawdust and corn pericarp, generate up to hundreds of tons annually, particularly in the northern regions of Thailand, presenting disposal difficulties and accumulating residue in the production system. Our goal is to address the gap between agricultural productivity and sustainability in the future by utilizing the diversity of fungal species and their capacity to convert organic waste into green composite materials with high strength and minimal environmental impact that can be applied as modern interior materials. At the same time, the integration of mycelium with agro-industrial waste presents an eco-efficient solution to reduce the strain on landfills, burning, and the environmental impact of various sectors [14]. Furthermore, the exploration of several different fungal species also provides insight into how they change the material characteristics of MBCs, increasing the range of applications across which they may be used [15].

In this paper, we delve into the basic principles of this sustainable innovation, describing the scientific research associated with MBCs, sourcing and preparing agro-industrial waste materials, selecting suitable fungal strains along with the production processes regarding green composite materials. At the same time, our research investigated the basic features of the generated MBCs, involving their physical, mechanical, and biological properties. Furthermore, we explore the future possibilities and potential challenges related to MBCs, offering a comprehensive perspective on their real-world applicability in the interior material sectors as well as their commercial potential. Through the advancement of knowledge regarding MBCs and their applications across interior material sectors, we hope to add to the current discourse on the sustainability of MBCs in this industry, encouraging a more ecologically conscious approach to manufacturing, while reducing waste and under-utilization of resources. Importantly, this study will serve as a guide towards sustainable innovation in green composite materials, offering a viewpoint for the future.

## 2. Materials and Methods

### 2.1. Source of Substrate and Initial Preparation

The main substrates in this study consisted of two different kinds of agro-industrial wastes, namely bamboo sawdust and corn pericarp, which were sourced from a bamboo sawmill and a corn processing factory located in Lampang and Chiang Mai Province, Thailand. Before testing, each substrate was sieved to obtain particles of the same size. Chips with a particle size of 1–5 mm were collected and used in this study. All selected substrates were dried in an oven, maintained at 60 °C for 24 h, until they were dried.

### 2.2. Mycelium Cultivation

The pure mycelia of four trimitic hyphal species (*Ganoderma fornicatum* CMU-NK0524; GF, *Ganoderma williamsianum* CMU-NK0540; GW, *Lentinus sajor-caju* CMU-NK0427; LS, and *Trametes coccinea* CMU-AM005; TC) along with one monomitic hyphal species (*Schizophyllum commune* CMU-S01; SC) were derived from the culture collection of the Research Center of Microbial Diversity and Sustainable Utilization (RCMU), and the Sustainable Development of Biological Resources Laboratory (SDBR-CMU), Faculty of Science, Chiang Mai University, Thailand. These species have been previously studied and have been reported to show potential for producing MBCs with several great properties, unique characteristics, and beautiful colors without the use of added pigments [11,16]. All fungal species were grown for 7 days at 30 °C on potato dextrose agar (PDA; Conda, Madrid, Spain).

### 2.3. Preparation of Substrate for Mycelium Culture

#### 2.3.1. Preparing an Inoculum for Fungal Mycelia

Sorghum grains were employed for mycelial inoculum production for each fungal species in this study. After cleaning, the sorghum was boiled at 100 °C for 20 min. Subsequently, 120 g of the boiled grains were placed in 250 mL conical flasks, sealed with cotton wool plugs, covered with aluminum foil, and autoclaved at 121 °C for 20 min for sterilization. After cooling for 24 h, approximately 1 × 1 cm mycelial pieces from each fungal species grown on the PDA medium were transferred into the flasks. The inoculated flasks were then incubated at 30 °C in darkness for 2 weeks until the sorghum grains were fully covered with fungal mycelia, suitable for use as inoculum [16].

#### 2.3.2. Preparing the Substrate for Mycelial Growth

Each type of dried substrate was mixed with additional nutrients, comprising 5% rice bran, 1% calcium carbonate, 2% calcium sulfate, and 0.2% sodium sulfate. The resulting mixtures were adjusted with reverse osmosis water (RO water) to achieve a total moisture content of around 60%, as measured by a moisture meter [17]. Eight hundred grams of the mixed substrate were placed into polypropylene culture bags measuring 3.50 inches wide × 12.5 inches long and sealed with cotton-plugged polyvinyl chloride pipe rings. All culture bags underwent autoclaving at 121 °C for 60 min. After cooling at room temperature for a full day, 5 g of each mycelial inoculum were transferred to the top of the substrate in each sterile culture bag. The inoculated bags were then incubated in the dark at room temperature (25 to 28 °C) for 21–30 days or until the substrates were completely covered by fungal mycelia [16].

### 2.4. Mold Design and Sterilization

**Figure 1:**
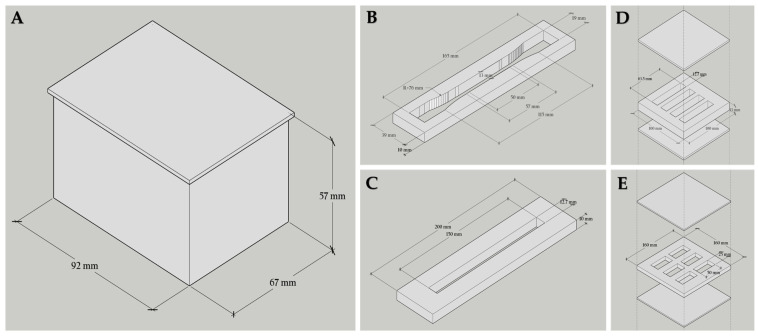
Designing molds for molding MBCs involves distinct examinations utilizing drawings created with Google SketchUp program version 8 for Windows: (**A**) molds for shaping MBCs in compression strength and water absorption tests, (**B**) molds for shaping MBCs in tensile strength tests, (**C**) molds for shaping MBCs in bending strength tests, (**D**) molds for shaping MBCs in impact strength tests, and (**E**) molds for shaping MBCs in soil burial tests.

The mold form employed for MBC production in each examination is illustrated in Figure 1. Plastic prism rectangular boxes measuring 92 × 67 × 57 mm^3^ served as molds for shaping composite samples in compression and water absorption tests. The molds for creating composite samples in tensile, bending, and impact tests were made from acrylic clear sheets, and cut into three different shapes in adherence to the American Society for Testing and Materials standard method (ASTM). These shapes included a dumbbell-shaped segment (165 × 19 × 10 mm^3^, neck 57 × 13 mm) and two rectangular shapes (150 × 12.7 × 10 mm^3^ and 63.5 × 12.7 × 13.0 mm^3^) [16]. For producing composite samples for the soil burial test, molds made from acrylic clear sheets were cut into rectangular shapes measuring 50 × 25 × 10 mm^3^, and modified according to the SR EN ISO 846/2000 standard [18] method for soil burial study [19]. Before their use in the composites forming process, all molds were sterilized by immersing in a 2% sodium hypochlorite solution for 5 min, followed by 2 rinses using sterile distilled water [20].

### 2.5. Mycelium-Based Bio-Composites Fabrication and Preparation for Testing

**Figure 2:**
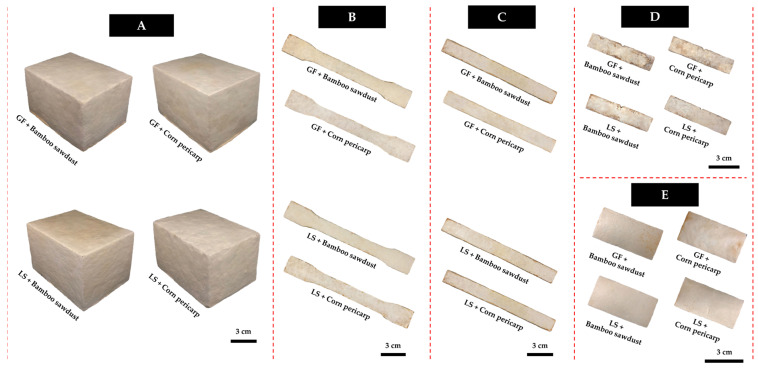
The obtained MBC samples in this study were derived from *Lentinus sajor-caju* and *Ganoderma fornicatum* in each substrate: (**A**) samples for compression and water absorption tests, (**B**) samples for tensile strength test, (**C**) samples for bending strength test, (**D**) samples for impact strength test, and (**E**) samples for soil burial test.

After complete colonization of the substrate by the fungal species, it was ground into small pieces and placed into the prepared molds. Each substrate underwent compression using a unidirectional cold press machine (Shop press ZX0901E-1, New Taipei, Taiwan) at a pressure of 0.5 MPa for 10 min, followed by incubation at 30 °C for 3 days in the dark. After this initial incubation period, the MBCs were removed from the molds and allowed to incubate for an additional 3 days within a plastic box until the mycelia completely covered the sides of the MBCs in contact with the mold. Following the curing period, the produced MBCs were dried at 70 °C for 24 to 72 h in a hot air oven until their mass stabilized [16]. The final MBC samples in each test (Figure 2) were investigated for their weight and sizes, and then kept in desiccators for further examination.

### 2.6. Determination of Physical Properties

#### 2.6.1. Moisture Content and Shrinkage

After the drying process, the moisture content and shrinkage of the MBCs in each variable were assessed. Moisture content was determined using the standard ASTM D 644 [21] method, which calculates the percentage mass loss: [(W1 – W2)/W1] × 100, where W1 is the initial sample mass, and W2 is the sample mass after drying. The shrinkage rate was determined based on wet and dry volumes, following the procedure outlined by Aiduang et al. [16]. The shrinkage percentage was expressed as (V1 − V2/V1) × 100, where V1 is the wet volume, and V2 is the dry volume. Ten replications were conducted for each treatment.

#### 2.6.2. Density Measurements

Density values were assessed using the dehydrated MBC samples employed in both compression and water absorption testing. The density of each specimen was determined by weighing the dried samples and calculating their volume by with the International Organization for Standardization (ISO) 9427 standard method [16]. The formula utilized for calculating the dry density of MBC samples was D = m/v, where D signifies the dry density in kg/m^3^, m denotes the mass of composites in kg, and v represents the volume of the sample in m^3^. This calculation was performed using the average values derived from ten test specimens for each treatment.

#### 2.6.3. Water Absorption and Volumetric Swelling

After being allowed to cool, the obtained MBCs were tested for water absorption following the standard method of ASTM C272/C272M-18 [15]. Before being tested, the initial mass and volumetric of the MBC samples were measured. Each specimen was then placed in the plastic container that contained the deionized water for a total of 96 h. Subsequently, specimens were weighed at 12, 24, 36, 48, 60, 72, 84, and 96 h to determine the changes in weight during each interval. The weight change was calculated using the equation described by Aiduang et al. [16]. Mass increasing (%) = [(W − D)/D] × 100, where W is the wet mass and D is the dry mass.

After a full 96 h of testing, the volumetric change of all specimens was measured using a digital vernier caliper. An increase in volumetric swelling was determined by applying the following equation to calculate the volume difference in relation to the initial volume: The volumetric swelling (%) = [V_2_ – (V_1_/V_2_)] × 100, where V_1_ is the sample’s initial volume and V_2_ is its expanded volume [22]. Ten replicates of each treatment were employed for the investigation.

#### 2.6.4. Thermal Degradation

Thermogravimetric analysis (TGA) of MBCs was carried out using a thermogravimetric analyzer (Rigaku: Thermo plus EVO2) from Tokyo, Japan. Each sample, weighing approximately 10 mg, was placed in an alumina crucible and subjected to heating in a nitrogen atmosphere, ranging from 25 to 600 °C at a rate of 10 °C/min [16].

### 2.7. Scanning Electron Microscope Observations

The surfaces and cross-sectional structures of the MBC samples obtained in this study were investigated. All dehydrated MBC samples were divided into small rectangular pieces, about 5 × 5 mm^2^, using a scalpel. The samples were then fixed to a 10 mm^2^ stub adapter using 2 mm^2^ double-sided carbon tape. After that, the samples were coated with gold for two minutes under a high vacuum mode. The prepared samples were subsequently investigated and captured on the image using a scanning electron microscope (SEM) JEOL JSM-5910 LV SEM (JEOL, Tokyo, Japan) with an initial voltage of 15 kV at the Science and Technology Service Center, Faculty of Science, Chiang Mai University, Chiang Mai, Thailand. To determine differences across treatments, the surfaces, and cross-sectional structures of the obtained images of MBCs in each variable were compared [16].

### 2.8. Determination of Mechanical Properties

#### 2.8.1. Bending Strength

The bending strength of the obtained MBC samples for each variable was evaluated following ASTM D 790-10 [21]. Employing a Hounsfield-H10Ks universal testing machine from New York, NY, USA, the bending test utilized a three-point bending setup with a cross-head speed of 2 mm/min and a clamp support distance of 40 mm. Force was applied to the specimen on the clamp support until it reached fracture. Stress was computed using the formula: σ = 3FL/2bh^2^, where σ represents the stress at the outer surface of the mid-span (MPa), F is the load (force) at the fracture point (N), L signifies the support span (mm), b is the width of the specimen (mm), and h represents the thickness of the specimen (mm). Each sample in each treatment underwent ten replications during the testing process.

#### 2.8.2. Compression Strength

In this investigation, the compression strength underwent testing following the guidelines of ASTM D 3501 [23]. The samples were placed on a Hounsfield H10Ks load workbench (New York, NY, USA), featuring a 10 kN capacity and a 1 kN load cell, all under normal conditions. The testing process maintained a regulated displacement speed of 5 mm/min. To determine compression strength, the resulting stress–strain curve was derived.

The load–displacement curve transformed into a stress–strain curve using the following formulas, enabling the calculation of compressive stress (σ) and strain (ε): Stress σ = F/A and Stress ε = ΔL/Lo, where F represents the compressive force (N), A is the original cross-section of the specimens (mm^2^), ΔL is the displacement of the loading surfaces (mm), and Lo is the original height of the test pieces (mm). Each treatment underwent investigation with ten replications, and the results are reported in MPa units.

#### 2.8.3. Impact Strength

The impact strength was determined through the Charpy impact test, following the ASTM D-256 standard [16]. In the testing process, samples were loaded into the machine and subjected to the pendulum until fracture occurred. The impact test enables the analysis of the material’s fracture and ductility in response to varying strain rates. Ten replications were performed on each sample in each of the treatments. Impact strength values were computed by dividing the energy required to fracture the sample (K) by its cross-sectional area (A), using the formula: Impact strength (kJ/m^2^) = K/A.

#### 2.8.4. Impact Strength

The tensile strength of MBC specimens was prepared and determined following the ASTM D 638-14 standard [24], employing a Hounsfield-H10Ks universal testing machine from New York, NY, USA. The testing used an elongation rate of 2 mm/min and a maximum force of 1 kN, conducted at room temperature with 40% relative humidity. Specimens were securely held in the grip of the tensile testing machine, and tension was applied until fracture occurred. The tensile stress was recorded in relation to the increase in strain, and the corresponding load and strain data were plotted on graphs. Each treatment used testing ten specimens to assess tensile behavior. The obtained data were analyzed to generate a stress–strain plot, indicating the tensile strength of the material.

### 2.9. Biodegradability Test

The biodegradability testing method, adapted from ISO 846/2000, was implemented under soil burial conditions using fine-grained natural active soil sourced from agricultural land in Chiang Mai Province, Thailand. Prior to testing, the soil underwent sieving to achieve a particle size of less than 2 mm [25]. MBC samples, carefully weighed with a digital laboratory scale (precision: 0.0001), were wrapped in a synthetic net before burial in soil. Ten replicates of the samples for each treatment were vertically buried in soil for a total of 90 days.

At intervals of 15, 30, 45, 60, 75, and 90 days, the samples were retrieved from the soil, cleaned to remove adherent soil, and dried at 70 °C until reaching a stable weight. The weight of the dried samples was measured, and the percentage weight loss was calculated using the formula: Total % weight loss = [(Initial wt. at day_0_ − Final wt. after day_15_)/Initial wt. at day_0_] × 100 [19].

### 2.10. Statistical Analysis

The data from each experiment were subjected to one-way analysis of variance (ANOVA) using the SPSS program version 16.0 for Windows. Subsequently, Duncan’s multiple range test was employed to identify significant differences (*p* ≤ 0.05) among the mean values.

## 3. Results and Discussion

### 3.1. Determination of Physical Properties

#### 3.1.1. Moisture Content and Shrinkage

**Figure 3:**
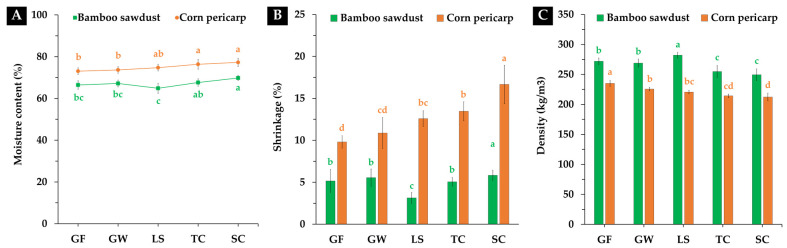
The moisture content (**A**), average shrinkage (**B**), and density (**C**) of the MBCs obtained in this study. The data are expressed as means with error bars representing the ± standard deviation. In the same experiment of each substrate type (depicted in the same color), different letters indicate significant differences according to Duncan’s multiple range test (*p* ≤ 0.05).

Typically, freshly produced MBCs tend to exhibit a relatively high moisture content during the initial manufacturing phase, attributed to the mycelium requires water for growth and to bond with the substrate [26]. In this investigation, the initial moisture content of the MBCs ranged from 66.44% to 77.27% on a wet-mass basis, displaying variations based on fungal species and substrate types, as depicted in Figure 3A. Specifically, the moisture content of MBC samples derived from bamboo sawdust ranged from 66.44% to 69.83%, while those from corn pericarp exhibited levels between 73.09% and 77.27%. Typically, the moisture content at this stage varies in the range of 59–80% by weight, depending on the production process [16,23,27,28]. These findings align with prior research indicating that the moisture content of MBCs is influenced by fungal species, substrate type, and growth conditions, which are associated with enzyme activities during fungal growth on the substrate [16,26,29]. Additionally, earlier studies have suggested that elevated moisture content may lead to shrinkage in mycelium-based composites, impacting their dimensional stability [23,30]. This becomes particularly crucial for interior material applications when precise dimensions are required.

Drying shrinkage is an essential part of each material’s volumetric stability [31]. The outcomes of this study revealed variations in the shrinkage values of the MBCs based on different parameters Figure 3B. Specifically, MBC samples produced from bamboo sawdust exhibited lower shrinkage values compared to those produced from corn pericarp across all fungal species, registering shrinkage percentages of 3.14% to 5.83% and 9.80% to 16.66%, respectively. Notably, MBCs derived from *L. sajor-caju* with bamboo sawdust as the substrate demonstrated minimal shrinkage, while MBCs produced from *S. commune* using corn pericarp exhibited the highest contraction rates. These findings align with earlier research suggesting that the shrinkage rate of MBCs may vary depending on the substrate type and mycelium species used in manufacturing [16,23,32]. Moreover, the shrinkage rate was influenced by the moisture content in the material samples and the drying method employed [23,30]. Nevertheless, the obtained results were consistent with the shrinkage observed in MBCs from prior research, falling within the range of 2.78% to 17% [16,23,31,32,33,34]. Interestingly, MBC samples utilizing bamboo sawdust as the base material consistently displayed lower shrinkage rates compared to MBCs from several earlier studies [16,23]. This highlights the potential influence of substrate selection on minimizing shrinkage in MBCs.

#### 3.1.2. Density

The density values of the MBCs obtained in this study exhibited variations based on parameters in the fabrication process, as depicted in Figure 3C. The results distinctly show that different fungal species and substrate types led to varied density values of the MBCs, ranging between 212.31–282.09 kg/m^3^. Notably, MBC samples produced from bamboo sawdust (249.50–282.09 kg/m^3^) demonstrated higher density than those made from corn pericarp (212.31–235.07 kg/m^3^) across all tested fungal species. The maximum density was observed in MBCs produced from *L. sajor-caju* combined with bamboo sawdust, while MBCs produced from *G. fornicatum* showed optimal density when utilizing corn pericarp as a substrate. MBCs made from *S. commune* exhibited the lowest density in both substrates. These findings align with previous investigations highlighting the significant influence of substrate type and fungal species on MBC densities [11,24,30,35,36]. Notably, the density values obtained in this study fell within the range of 25–954 kg/m^3^, consistent with previously published research [15,24,30,37,38,39,40]. Additionally, numerous studies have reported various factors affecting MBC density, including substrate composition, substrate particle size, mycelium strain, growth conditions, growth time, post-processing techniques, mold used, and drying conditions [10,13,30,31]. Despite this variability, the density values of MBCs in this study were comparable to those of many traditional materials, such as synthetic materials (11–920 kg/m^3^) [16,41] and paper-based materials (36–1522.4 kg/m^3^) [42,43], suggesting their possible use in a variety of fields, especially as interior materials.

#### 3.1.3. Water Absorption and Volumetric Swelling

**Figure 4:**
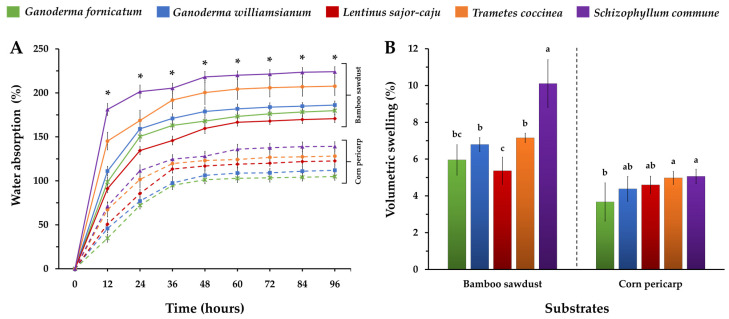
The water absorption abilities (**A**) and volumetric swelling levels (**B**) of the MBCs were obtained in this investigation. The presented data represents means, with error bars at each point indicating the ± standard deviation. In (**A**), “*” signifies a significant difference based on Duncan’s multiple range test (*p* ≤ 0.05) at each point. In the experiment for each substrate type (**B**), different letters denote significant differences according to Duncan’s multiple range test (*p* ≤ 0.05).

The water absorption ability of the MBCs obtained from bamboo sawdust and corn pericarp was assessed by immersing the MBCs in water for 96 h, as depicted in Figure 4A. The results indicated that MBCs made from bamboo sawdust exhibited a higher water absorption ability compared to those made from corn pericarp. The water absorption of bamboo sawdust MBCs increased rapidly within the initial 24 h and gradually stabilized after 48 h. Similarly, water absorption of corn pericarp MBCs exhibited an initial sharp increase within the first 36 h, followed by a slower stabilization after 60 h. After 96 h, it was observed that MBCs produced from bamboo sawdust displayed water absorption rates ranging between 170.70 and 224.08%, while MBCs produced from corn pericarp exhibited water absorption capacities around 104.89 and 139.22%. This study further identified that MBCs produced from S. commune displayed the highest water absorption ability, while those made from *L. sajor-caju* exhibited the lowest among the bamboo sawdust composites, and MBCs made from *G. fornicatum* had the lowest rate among the corn pericarp composites.

The water absorption ability (104.89–244.08%) of the MBCs in this study fell within the ranges reported in previous studies (24.45–560%) when submerged in water over 24–192 h [16,23,24,39,44]. Generally, MBCs were characterized as hydroscopic materials, and their water absorption ability was influenced by many parameters [16,41]. First, this study found an inverse relationship between the density of MBCs and their water absorption ability, aligning findings from prior studies, indicating a decrease in water absorption capacity with an increase in composite density [15]. Second, variations in water absorption abilities were linked to differences in the chemical components of the composites, with bamboo sawdust (40.0–47.7% dry mass basis) containing a higher cellulose content than corn pericarp (15.3–22.5% dry mass basis), influencing the high-water absorption ability observed in this investigation [16,45,46,47,48,49]. This characteristic is often associated with a higher cellulose component, indicating a larger number of accessible hydroxyl groups [16,41,50]. Third, the dense coverage of fungal hydrophobic mycelium on the material surface was found to improve the water absorption resistance of MBC samples [24,51]. Additionally, it was observed that the water absorption ability of MBCs decreased when smaller particle-sized substrates were used [52].

Despite these insights, the water absorption capacity of MBCs remains an area of concern in comparison to conventional synthetic materials. However, this suggests a need for ongoing improvements to fully unlock the potential of MBCs. Nevertheless, the substantial water absorption capacity of MBCs may not adversely affect industries like interior design [53], especially when used for insulation and acoustic boards, along with photo frames. Future enhancements in this aspect could further broaden the possibilities and applications for MBCs.

The volumetric swelling of MBCs in this study was observed after 96 h of water soaking, ranged from 3.67% to 10.10% (Figure 4B). Notably, MBCs crafted from bamboo sawdust exhibited a higher volumetric swelling rate (5.37 to 10.10%) compared to those made from corn pericarp (3.67 to 5.06%). Among the MBCs, those produced from corn pericarp and *G. fornicatum* displayed the lowest volumetric swelling rates, while MBCs from *S. commune*, across both substrate types, exhibited the highest volumetric swelling. The findings found distinct volumetric swelling patterns in MBC samples based on the substrate type and fungal mycelium species, directly influenced by the material’s water absorption behavior. This study attributed the increased swelling to the material’s heightened ability to absorb water, aligning with several previous research projects [22,29,54]. Importantly, the volumetric swelling of MBCs in this study was within the range reported in earlier studies (0.28 to 21%), with the extent of swelling varying according to the MBCs distinct water absorption capabilities [22,29,55].

#### 3.1.4. Thermal Degradation

**Figure 5:**
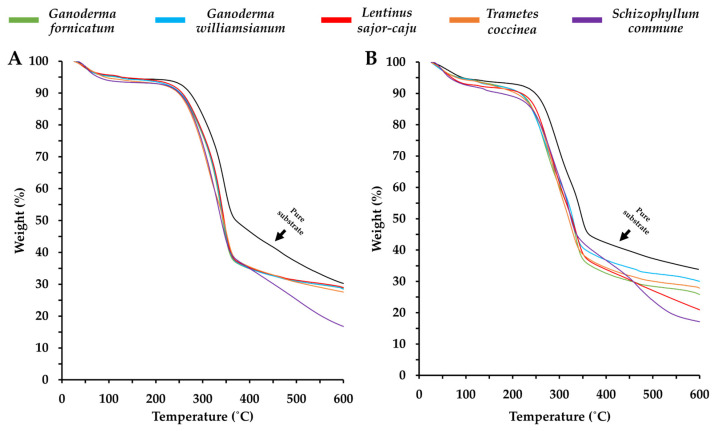
Thermogravimetric analysis of MBCs produced in this study utilizing a combination of each fungal species with bamboo sawdust (**A**) and corn pericarp (**B**).

Figure 5 illustrates the thermogravimetric analysis results for the MBCs obtained in this study. The degradation behaviors observed align with previous research, identifying three distinct phases of mass loss. The initial phase involves the evaporation of free and chemically connected moisture, resulting in a mass loss of approximately 5 to 9%, occurring between 25 to 125 °C. The second phase shows degradation with around 70% mass loss, observed at temperatures ranging from 200 to 325 °C for MBCs produced from bamboo sawdust and 180 to 300 °C for those produced from corn pericarp. The third stage, related to decomposition, occurs at temperatures ranging from 350 to 375 °C for bamboo sawdust MBCs and 325 to 375 °C for corn pericarp MBCs. All degradation characteristics of the obtained MBCs demonstrated behaviors similar to the level regarding the rate of thermal degradation for each lignocellulosic substrate utilized, albeit with a faster rate of weight decrease.

According to several previous investigations, MBC materials typically exhibit three stages of change in their thermal degradation behaviors, following the composition of the substrate [24,56]. Investigations involving TGA have demonstrated that cellulose, hemicellulose, and lignin decompose at varying temperatures. In general, cellulose degrades at a higher temperature range (300–400 °C) than hemicellulose (220–315 °C), whereas lignin degrades across a wide range of temperatures (150–900 °C) [47,57]. Our research revealed distinct thermal degradation behaviors in MBCs made from bamboo sawdust and corn pericarp, likely associated with the chemical composition of the substrates. Typically, bamboo sawdust has an estimated dry mass basis composition of 40 to 47.7% cellulose, 21.9 to 31% hemicellulose, and 21 to 24.9% lignin, while corn pericarp consists of 15.3 to 22.5% cellulose, 23.7 to 40.4% hemicellulose, and 2.9 to 4.7% lignin on a dry mass basis [45,46,47,48,49], potentially impacting their degradation behaviors. Simultaneously, the weight loss of both pure substrates was slower than that of colonized substrates, indicating that fungal colonization renders the substrate more susceptible to thermal degeneration [24].

However, the obtained thermal degradation values were within the ranges reported in other studies, where the mass loss during the first stage ranged between 5–10% at temperatures from 25 °C to 200 °C. Subsequently, the second stage involves significant degradation (approximately 70% weight loss) occurring between 200 to 375 °C, followed by the third stage involving the decomposition process starting from 350 °C [16,30]. Upon conducting a comparative analysis, it is noteworthy that the MBCs produced in this study demonstrated overall thermal degradation behaviors like those observed in various paper-based products (250 to 350 °C) and synthetic foams (250 to 475 °C) [16,58,59,60,61]. Additionally, the thermal characteristics of the MBCs align with those of green composite materials known to degrade above 200 °C [62]. This congruence in thermal degradation behaviors emphasizes the potential applicability of MBCs across diverse industries, as their thermal properties closely resemble those of established paper-based products, synthetic foams, and green composite materials. Such versatility positions MBCs as promising materials with thermal stability suitable for a wide range of applications.

### 3.2. Scanning Electron Microscope Observations

**Figure 6:**
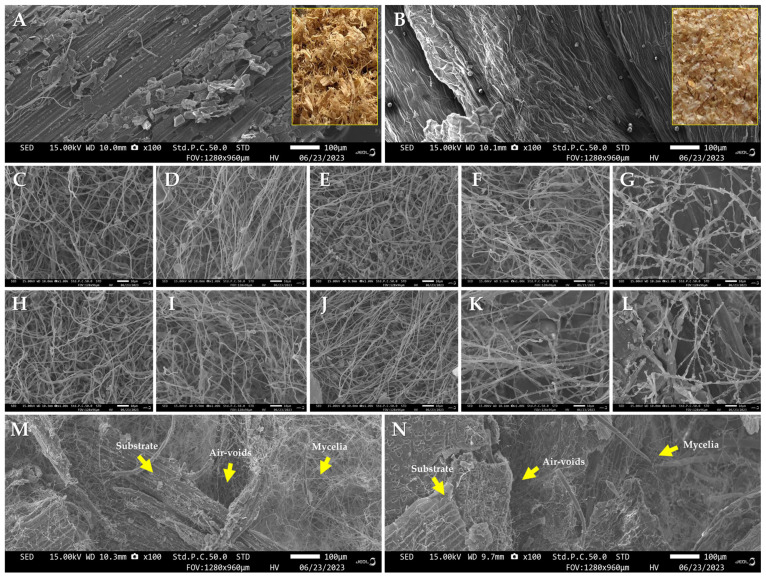
The scanning electron microscopic images of MBCs obtained in this study: The original bamboo sawdust (**A**) and corn pericarp (**B**) particles. The surfaces of MBCs produced from bamboo sawdust combined with *G. fornicatum* (**C**), *G. williamsianum* (**D**), *L. sajor-caju* (**E**), *T. coccinea* (**F**), and *S. commune* (**G**). The surfaces of MBCs produced from corn pericarp combined with *G. fornicatum* (**H**), *G. williamsianum* (**I**), *L. sajor-caju* (**J**), *T. coccinea* (**K**), and *S. commune* (**L**). The cross-sectional structure of MBCs derived from bamboo sawdust combined with *L. sajor-caju* (**M**) and corn pericarp combined with *G. fornicatum* (**N**). Yellow arrows represented the internal structure of the MBCs, consisting of air-voids, fungal mycelia, and substrate.

The morphological features of the MBCs obtained in this study, both in cross-sectional structure and surface areas, were examined and compared with the raw materials (non-colonized substrate), as illustrated in Figure 6. Initially, the surfaces of bamboo sawdust and corn pericarp appeared exceptionally pure and smooth on the original substrate particles (Figure 6A,B). After mycelial colonization, a fungal mycelial network covered the previously smooth surface of the substrate particles. Upon visual assessment of SEM micrographs, it became evident that the surfaces of all obtained MBCs were enveloped by fungal mycelia, with variations based on the types of mycelium binder networks, each characterized by distinct mycelia densities (Figure 6C–L). Surfaces covered by trimitic hyphal species (*G. fornicatum, G. williamsianum, L. sajor-caju*, and *T. coccinea*) exhibited a higher mycelia density compared to monomitic species (*S. commune*). Notably, MBCs created from *L. sajor-caju* demonstrated a greater mycelia density than other fungal species when grown on the surface of bamboo sawdust, whereas *G. fornicatum* displayed a higher mycelia density when covered on the surface of corn pericarp. This difference directly impacts the physical and mechanical properties of the MBC materials, particularly their density, bending, compression, tensile, and impact strength, which are influenced by the types of mycelium binder networks and mycelial density [16].

Examination of the sectional areas of MBCs revealed that fungal mycelia fused substrate particles together through various mycelial networks, exposing air-voids within the composites (Figure 6M,N). These morphological characteristics align with prior research studies, indicating that the raw materials surface was covered by a fibrous network of fungal hyphae after mycelium colonization [16,24,53,63]. Typically, fungal growth and colonization on substrates involve a combination of apical extension of hyphal tips and the generation of new hyphal tips through branching. An important aspect is that while extension occurs only at the tip at a linear and constant rate, the frequency of branching results in an exponential growth pattern of mycelial biomass, particularly in the initial stages of the vegetative phase. This hyphal growth pattern empowers filamentous fungi to effectively penetrate lignocellulosic substrates. The cell wall structure at the tip and the branching of the mycelium contribute to a sturdy and solid structure. Additionally, hydrolytic enzymes are secreted at the hyphal tip, enhancing their efficiency, and facilitating penetration into lignocellulosic substrates [64]. This penetration improves the accessibility of nutrients within the particles as a fundamental mode of fungal growth and colonization acts like a glue, binding the particles of the lignocellulosic substrate together while presenting air-voids into these composite systems [16,63]. However, their filamentous growth mode’s effectiveness in substrate colonization also depends on substrate characteristics such as stiffness, surface, volume, and chemical components [65].

### 3.3. Determination of Mechanical Properties

#### 3.3.1. Bending Strength

**Figure 7:**
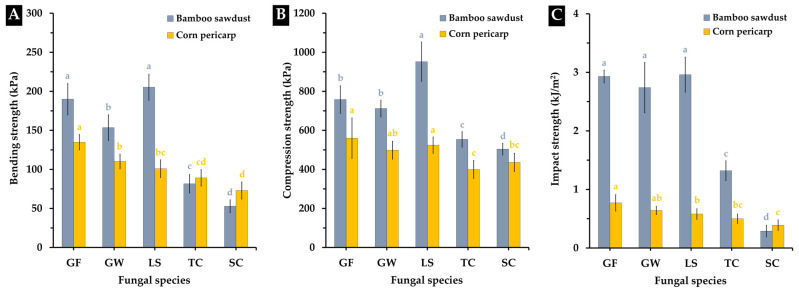
Bending (**A**), compression (**B**), and impact strengths (**C**) of MBCs produced from a combination of each fungal species with bamboo sawdust and corn pericarp. The presented data consists of means, with error bars at each point indicating the ± standard deviation. Significance in differences within the same experiment for each substrate type (depicted in the same color) is determined by Duncan’s multiple range test, where distinct letters denote statistical significance (*p* ≤ 0.05).

The bending strength of MBC specimens, produced from the combination of each fungal species with sawdust from bamboo and corn pericarp, is depicted in Figure 7A. The overall bending strength levels of the MBCs ranged from 52.79 to 205.08 kPa, displaying variations based on different factors. Notably, MBCs from *L. sajor-caju* combined with bamboo sawdust exhibited the highest bending strength, while MBCs from *G. fornicatum* demonstrated peak bending strength when cultivated on corn pericarp (134.86 kPa). In contrast, MBCs derived from *T. coccinea* and *S. commune* exhibited a lower bending strength compared to other fungal species. These findings align with much prior research, which found that differences in bending strength levels in MBC materials are often influenced by multiple manufacturing details. The different growth rates, density, mycelia network, and bonding capabilities of each fungal strain contribute to varied bending properties [16,26,41,66]. Trimitic fungal species, characterized by thick-walled, dense, and hard hyphae, generally result in MBCs with higher bending strength than monomitic and dimitric species [24,41]. Moreover, the bending strength of MBCs is linked to the substrate type and the pressing method employed. Substrates with different compositions and particle characteristics interact uniquely with mycelium, affecting the overall strength of the composite. The pressing action caused differences in the mechanics between the fungal mycelium and substrate, which improved the MBCs elasticity and strength [24,30]. Additionally, prior research suggested that density plays a role, with higher density often correlating with increased bending strength, although there may be trade-offs with other properties like weight and porosity [44,67].

However, the obtained MBC values in this study were within the range reported in previous investigations (50–4400 kPa), comparable to expanded polystyrene foam (75–3000 kPa) [16,41,68]. This suggests the viability and potential of MBCs as substitute materials for foam in the interior design sector soon, particularly for non-structural or semi-structural elements that do not require load-bearing materials or for specific structural elements that require minimal load-bearing forces [69].

#### 3.3.2. Compression Strength

Compression strength plays a crucial role in the mechanical properties of MBC materials when employed in interior design applications, as the material may be subjected to weight-bearing requirements during transportation and utilization in some fields [26]. This study unveiled variable compressive strength values based on the type of fungal mycelium and substrates, suggesting potential applications across diverse interior material sectors. The compressive strength of MBCs in this study ranged from 400 to 952 kPa (Figure 7B). Notably, MBCs created from bamboo sawdust (504 to 952 kPa) exhibited superior compressive strength compared to those from corn pericarp (400 to 560 kPa) across all utilized fungal mycelium. Specifically, MBCs produced from *L. sajor-caju* combined with bamboo sawdust displayed the highest compressive strength among all obtained MBCs. When corn pericarp was employed, MBCs showed commendable compressive strength, especially when using *G. fornicatum*, *G. williamsianum*, and *L. sajor-caju* as biopolymers. However, MBCs produced with fungal mycelium from *T. coccinea* and *S. commune* demonstrated comparatively lower compressive strength. These findings not only illustrate a variety of compressive strength levels but also align with previous studies, indicating that the compression strength of MBC materials typically falls within the 30 to 4400 kPa range [23,30,37,39,44,70,71].

The disparities in compression strength values of MBC materials typically result from a combination of factors, including compositional variations, mycelium growth, material density, porosity, post-processing techniques along with degree of pressing. These factors are all related to the different substrate kinds and fungal species that have been employed in the manufacturing process [30,44,72]. Furthermore, the application of pressure during production was identified as a contributing factor that effectively enhanced the compressive strength of MBCs [16]. Despite these variations, the obtained MBCs demonstrated compression strength levels akin to various materials commonly utilized in contemporary interior design applications, such as synthetic foams, natural materials, and paper-based materials [16,44,67]. This positions them as promising alternatives within this field.

#### 3.3.3. Impact Strength

Ten different MBC forms exhibited different behavior when subjected to an action force (Figure 7C). These variations were attributed to differences in the types of substrates used and the fungal mycelium employed during manufacture. The impact strength levels of the MBCs in this study were observed to fall within the range of 0.29 to 2.96 kJ/m^2^. Notably, MBCs created from bamboo sawdust in combination with trimitic fungal species demonstrated many times higher impact resistance than MBCs made from corn pericarps, particularly those derived from *G. fornicatum*, *G. williamsianum*, and *L. sajor-caju*. The MBCs with the highest impact strength were derived from *L. sajor-caju* combined with bamboo sawdust (2.96 kJ/m^2^), while effective impact strength in corn pericarp-derived MBCs was achieved with *G. fornicatum* (0.77 kJ/m^2^) as the binder. In contrast, the utilization of *S. commune* in MBC production resulted in a lower impact strength for the final composite material in both substrate types.

The difference in impact strength observed among MBC materials can be primarily attributed to the fungal mycelium species and substrate type. These factors are linked to the mycelium binder network system, fiber structure, and matrix strength. In general, MBCs produced from trimitic fungal species exhibited a higher impact strength due to the presence of a network characterized by stiff, dense, and thickly walled hyphae, which are similar to the bending and tensile behavior observed [16]. Furthermore, the diversity in impact strength across different MBC forms may be influenced by various additional factors, including load transfer performance, break propagation resistance, bonding toughness, fiber distribution, and geometric considerations [16,73,74]. Yet, the impact strength levels obtained in this study align with previously reported values ranging between 0.21–2.70 kJ/m^2^ [16]. Importantly, these values fall within the range observed for many traditional materials, particularly foam-based (0.001–5 kJ/m^2^) and paper-based (2–12 kJ/m^2^) materials [30,66,75,76,77]. This underscores the significant potential of the MBC materials for applications in modern interior elements.

#### 3.3.4. Tensile Strength

**Figure 8:**
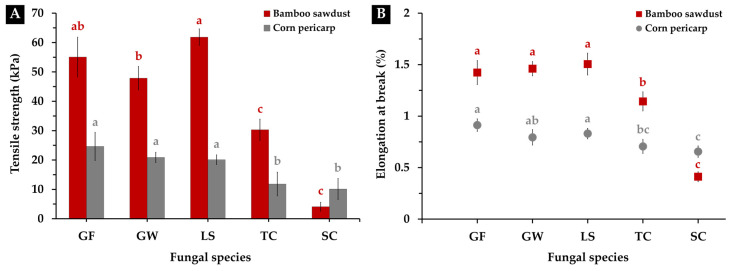
Tensile strength (**A**) and elongation at break (**B**) of MBCs produced from a combination of each fungal species with bamboo sawdust and corn pericarp. The presented data shows means, and error bars at each point indicate the ± standard deviation. Significance in differences within the same experiment for each substrate type (depicted in the same color) is determined by Duncan’s multiple range test, where distinct letters denote statistical significance (*p* ≤ 0.05).

Tensile strength and elongation at break are crucial mechanical properties for evaluating the performance of interior materials [78]. In this study, the measured tensile strength of MBCs exhibited a diverse range of values, influenced by the substrate type and fungal mycelium used as a biomatrix, as depicted in Figure 8A. MBCs crafted from bamboo sawdust displayed tensile strength levels ranging from 4.10 to 61.85 kPa, while those made from corn pericarp had values between 10.13 to 24.63 kPa. Among the investigated combinations, MBCs from bamboo sawdust paired with *L. sajor-caju* showcased the highest tensile strength, with no statistically significant difference observed compared to those from *G. fornicatum*. Simultaneously, utilizing the fungal mycelium of *G. fornicatum*, *G. williamsianum*, and *L. sajor-caju* as a binder with corn pericarp as the substrate resulted in MBCs demonstrating good tensile qualities. Conversely, MBCs employing *S. commune* and *T. coccinea* in manufacturing exhibited lower tensile attributes than other fungal species in both substrate types.

These findings align with the theory of earlier studies on MBC production, indicating that the tensile strength of MBCs is influenced by various factors associated with substrate type, mycelium binder network structure, and the pressing process [30,41,44,67]. Generally, the particle size and structure of each substrate material can impact the porosity and overall structure of MBCs, thereby influencing tensile strength and other mechanical properties [79]. At the same time, using trimitic fungal species in MBC production led to higher tensile strength, attributed to their thick-walled, dense, and hard hyphae, contributing to the stiffness of the composite material [16,26,31,41,44]. Moreover, previous research has also suggested that the pressing process enhances tensile strength for MBCs, improving the interconnectivity of the mycelial network with substrate particles within the composites. This enhanced interconnectivity promotes a more uniform distribution of fungal mycelium throughout the material, enhancing load-bearing capabilities [24,40,80].

The elongation at break of the MBCs obtained in this study followed a similar trend to their tensile characteristics, with values ranging from approximately 0.41% to 1.51% for MBCs produced from bamboo sawdust and about 0.66% to 0.91% for those made from corn pericarp (Figure 8B). Notably, MBCs generated from fungal mycelium with a trimitic hyphal system demonstrated strong elongation at break. The correlation between the elongation at break value and tensile strength in MBC materials is generally attributed to the material’s overall structural characteristics and the interplay of various factors during its formation [24,26]. Elongation at break and tensile strength are interconnected mechanical properties that reflect how a material responds to stress and deformation [81].

Nevertheless, the results obtained in terms of tensile strength and elongation at break were within the established range reported in previous research studies, typically around 10–1550 kPa and 0.7–4.7%, respectively [16,24,30,44]. Additionally, upon comparison with traditional materials, these characteristics demonstrated a level of compatibility with certain materials commonly employed in interior element applications (e.g., silicone foam and paper honeycomb) [68,82,83]. Looking ahead, further enhancements in these features could potentially broaden the scope of applications for MBCs, opening opportunities for even more diverse uses in the future.

### 3.4. Biodegradability Test

**Figure 9:**
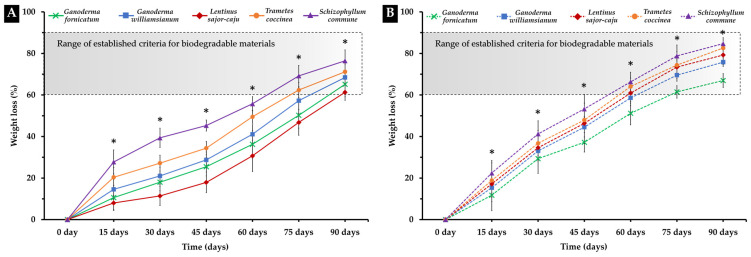
The cumulative percentage of weight loss for the MBC samples used in this investigation that were buried in soil. The weight loss percentage of the MBCs derived from bamboo sawdust (**A**) and corn pericarp (**B**). The data is presented as means, and error bars at each point indicate the ± standard deviation. The “*” symbol indicates a significant difference based on Duncan’s multiple range test (*p* ≤ 0.05) at each point.

The percentage weight loss of the MBC samples buried in soil for 90 days are shown in Figure 9. The results revealed variations based on the mycelium species and the type of substrate utilized. MBCs produced from bamboo sawdust and corn pericarp, in combination with each mycelium species, exhibited a percentage of weight loss ranging between 61.31 to 84.71% after being buried for 90 days. Specifically, MBC specimens derived from corn pericarp showed a faster rate of degradation compared to those produced from bamboo sawdust. Nevertheless, all MBCs exhibited degradation rates surpassing 60%, falling within the generally recognized standard for biodegradable materials. These findings align with prior studies indicating the substantial influence of different substrate types and fungal species on material degradation in MBCs [22,84,85,86]. However, variations in degradation rates may be influenced by additional factors, including material composition, strength, physical and chemical properties, microbial activity (fungi and bacteria), and durability to weathering [30].

In general, the percentage weight loss of the MBC materials ranged between 13.19–70% when buried in soil for 1–4 months [22,30,85,86]. These results align with established criteria for biodegradable materials, defining biodegradability within the range of 60–90% over of 3–24 months [87]. This consistency with accepted standards underscores the potential of MBCs as environmentally friendly materials that comply with established biodegradability criteria.

### 3.5. Determination of Mechanical Properties

Mycelium-based composite materials have gained attention as sustainable alternatives for interior design applications, offering several advantages when compared to traditional materials like synthetic foams and paper-based products [11,44,88]. Here is a comparison of their obtained overall properties (Table 1). Our current research revealed that the majority of MBCs developed had overall physical, mechanical, and biological properties that were comparable to many paper- and foam-based materials used in the interior design sector nowadays.

Regarding physical properties, the MBCs obtained in this study fell within the range observed in previous studies. Specifically, the density levels of the obtained MBCs were comparable to those of paper-based materials, such as corrugated cardboard (98.3–691 kg/m^3^), paperboard (200–800 kg/m^3^), and paper honeycomb (10–321 kg/m^3^). However, their density was higher than that of many synthetic foams. This characteristic positions MBCs as an appealing choice for sustainable interior material, offering advantages in terms of strength and load-bearing qualities. In terms of shrinkage and water absorption, the obtained MBCs were found to be most similar to paper-based materials, although they demonstrated higher values than synthetic foam materials. The volumetric swelling after water absorption revealed that MBCs (3.67–10.10%) exhibited greater swelling than synthetic materials but remained within the range observed for paper-based materials (0.05–9%). Interestingly, in terms of thermal degradation, the MBCs (180–325 °C) in this study fell within the range observed for both synthetic foams (250–475 °C) and paper-based materials (250–350 °C).

Considering mechanical properties, the obtained MBCs exhibit similarities with those from prior research studies and demonstrate comparability with various conventional materials. In terms of compression strength, the obtained MBCs (400–952 kPa) surpassed levels observed in expanded polystyrene foam (100–180 kPa) and silicone foam (8–170 kPa). They exhibited similarities to foamed glass (400–3000 kPa), extruded polystyrene foam (200–700 kPa), phenolic formaldehyde resin foam (200–550 kPa), polyurethane foam (2–48,000 kPa), corrugated cardboard (7.94–1345.7 kPa), paperboard (400–10,000 kPa), and paper honeycomb (250–94,000 kPa). However, in comparison to polypropylene foam (31,190–48,290 kPa), they still demonstrated lower compression strength. Regarding bending strength, the MBCs (52.79–205.28 kPa) showed relatively lower values compared to many traditional materials but remained comparable to expanded polystyrene foam (75–3000 kPa), a widely used material in interior element applications. The impact strength of the obtained MBCs (0.29–2.96 kJ/m^2^) was within the range of various synthetic foams and paper materials, such as foamed glass (0.001–5 kJ/m^2^), extruded polystyrene foam (0.16–2.14 kJ/m^2^), phenolic formaldehyde resin foam (0.26–1.63 kJ/m^2^), polypropylene foam (0.02–3 kJ/m^2^), polyurethane foam (0.38–1.2 kJ/m^2^), and paperboard (2–4 kJ/m^2^). Notably, their impact strength exceeded that of expanded polystyrene foam (0.22–0.245 kJ/m^2^) but remained lower than paper honeycomb (4.8–12 kJ/m^2^). In terms of tensile and elongation properties, the MBCs exhibited lower values compared to many traditional materials. However, their tensile strength aligned with silicone foam (55.2–2800 kPa) and paper honeycomb (49.3–22,770 kPa), while the elongation at break were within the range of extruded polystyrene foam (1–70%), phenolic formaldehyde resin foam (0.2–15.7%), as well as paperboard (1–3.5%). This described comparison highlights the many mechanical properties of MBCs, indicating them as materials with special qualities that fall within the category of traditional options.

Nevertheless, the distinctive advantage of MBCs lies in their biodegradability, setting them apart from synthetic foam materials. This characteristic provides a significant advantage when applied in modern interior materials, as they can decompose in the environment at the end of their useful life. Additionally, their biodegradability is comparable to that of paper materials, underscoring their potential to evolve into advanced interior materials for sustainable, long-term use in the future.

### 3.6. Challenges, Future Perspectives, and Development Approaches in Terms of Applications and Modern Interior Prototypes

Addressing challenges and adopting strategic development approaches are crucial for the successful application of MBCs in modern interior elements [124]. Present research highlights the promising potential of MBCs derived from bamboo sawdust and corn pericarp, showcasing comparable properties to traditional materials like synthetic foams and paper-based materials.

**Figure 10:**
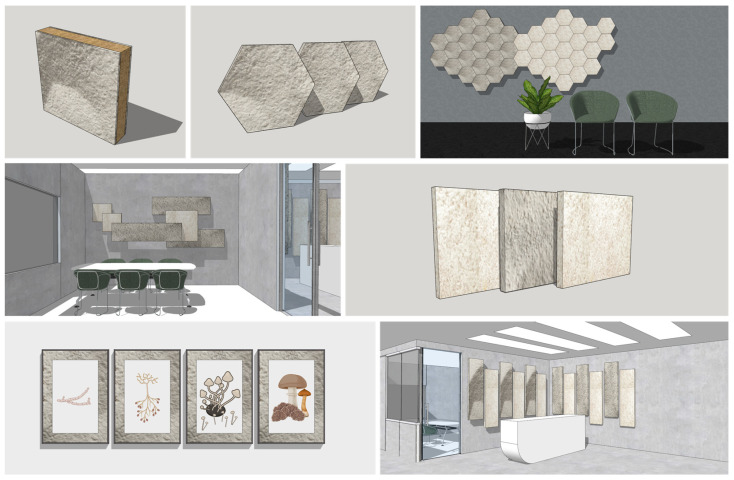
The possibility for next-generation environmentally friendly interior elements made from MBC materials, contributing to environmental protection within a circular economy system.

To unlock the full potential of MBCs, challenges such as standardizing production processes, ensuring consistency, and achieving material performance and durability standards must be addressed. Balancing degradation and strength are key, along with overcoming cost-effective production challenges and scaling up manufacturing [16,35,67,125,126]. At the same time, building awareness among consumers and industries about the benefits of mycelium-based interior materials is crucial for market adoption. Strategic communication, along with creative and unique product prototypes, enhances the appeal to customers (Figure 10) [126]. Moreover, ongoing research in material engineering, incorporating additives, and harnessing biotechnological advances hold promise for developing MBCs with enhanced properties. Continuous development through collaborative research, interdisciplinary cooperation, increased investment, and integration into circular economy models ensures the sustainability and commercial viability of MBC materials [127].

Although MBCs show comparable properties to traditional materials, challenges in standardization, performance, cost-effective production, and market adoption require further research. Ongoing efforts in material engineering and biotechnological advancements are suggested to enhance MBC properties, emphasizing a continuous development approach for sustainability and economic value.

## 4. Conclusions

The research investigated the various physical, mechanical, and biodegradable properties of MBCs derived from different fungal species and substrate types. In terms of moisture content and shrinkage, MBCs displayed variations influenced by fungal species and substrate types. Notably, bamboo sawdust-based MBCs exhibited lower shrinkage and moisture content compared to those derived from corn pericarp. Density values ranged from 212.31–282.09 kg/m^3^, showing variations based on fungal species and substrate types, with bamboo sawdust-based MBCs generally having higher density. Water absorption and volumetric swelling were assessed, revealing that bamboo sawdust MBCs had higher water absorption than corn pericarp MBCs, which also led to larger swelling values. Despite concerns about water absorption capacity, the study highlights potential applications in interior design industries where there is no risk of contact with water. Thermal degradation analysis demonstrated behaviors consistent with prior research, with MBCs exhibiting stages of mass loss aligned with the composition of the substrates. The obtained thermal degradation values fell within the ranges reported in other studies, emphasizing the potential applicability of MBCs in diverse industries, given their thermal stability. In terms of the MBCs mechanical properties, including bending, compression, impact, and tensile strengths, were evaluated. Bending and compression strengths varied based on fungal species and substrate types, with bamboo sawdust-based MBCs generally exhibiting superior strength. Impact strength levels varied, influenced by fungal species and substrate types, but overall, MBCs demonstrated values comparable to traditional interior materials. Tensile strength and elongation at break were influenced by substrate type and fungal species, with trimitic species generally contributing to higher tensile strength. In terms of biodegradability, the assessment revealed that MBCs, irrespective of fungal species and substrate types, exhibited degradation rates surpassing 60% after 90 days of burial in soil, aligning with biodegradability standards.

In summary, this research provides a comprehensive analysis of MBCs, demonstrating their potential as sustainable alternatives for modern interior materials. The study emphasizes their comparable physical and mechanical properties to traditional materials, particularly MBCs produced from *L. sajor-caju* and *G. fornicatum*. With the added advantage of biodegradability, positioning MBCs as promising candidates for various applications in the future. Despite challenges in standardization, production cost, and market adoption, ongoing research in material engineering, and biotechnological advancements promises enhanced MBC properties. Strategic development, collaborative research, interdisciplinary cooperation, and integration into circular economy models are essential for the sustainability and commercial viability of MBC materials. Addressing these challenges will unlock the full potential of MBCs as sustainable alternatives in modern interior materials.

## Figures and Tables

**Table 1:** Table 1 presents a comparison of the properties of the MBCs developed in this study with those reported in previous studies, along with synthetic foam- and paper-based products commonly used in interior design applications. The data have been modified and adapted from Jones et al. [41] and Aiduang et al. [16,30].

	Types	Properties *										
		D (kg/m^3^)	AS (%)	WP (%)	VS (%)	TD (%)	CS (%)	BS (%)	IS (%)	TS (%)	EAB (%)	B (%)
Mycelium-Based Bio-Composites	This study	212.31–281.33	3.14–16.66	104.89–224.08	3.67–10.10	180–325	400–952	52.79–205.28	0.29–2.96	4.1–61.85	0.41–1.51	61.31–84.72
	Previous studies	25–954	6.2–16.31	24.5–560	0.28–21	225–375	30–4400	50–4400	0.21–2.7	10–1550	0.7–4.7	19–70
Synthetic Foams	Foamed Glass	100–400	0.01–2	0.8–11	–	300–450	400–3000	^<^ 300–3000	0.001–5	^<^ 660–1590	^<^ 1.7–11	NB
	Expanded Polystyrene foam	11–32	0.2–5	0.03–9.00	–	318–440	100–180	75–3000	0.22–0.245	^<^ 80–170	^<^ 5–13.4	NB
	Extruded Polystyrene foam	28–50	0.2–1.5	0.25– 0.9	–	318–440	200–700	500–1000	0.16–2.14	^<^ 200–520	1–70	NB
	Phenolic Formaldehyde Resin foam	35–120	0.1–1	1–15	–	270–475	200–550	^<^ 380–780	0.26–1.63	^<^ 190–460	0.2–15.7	–
	Polypropylene foam	895–920	1.0–2.5	0.01–0.8	–	360–460	^<^ 31,190–48,290	^<^ 20,000–23,200	0.02–3	^<^ 9000–41,400	^<^ 2.4–900	NB
	Polyurethane foam	30–100	0.59–2	0.3–20.0	–	278–379	2–48,000	^<^ 210–56,500	0.38–1.2	^<^ 80–103,000	^<^ 3.2–760	NB
	Silicone foam	104–164	0.15–1	0.2–12.0	–	250–350	8–170	–	–	55.2–2800	^<^ 49–300	NB
Paper-Based materials	Corrugated Cardboard	98.3–691	5.36–13.45	98–161	0.52–4	260–347	7.94–1345.7	^<^ 770–2510	–	^<^ 400–3000	^<^ 6.7–7.7	80–88
	Paperboard	200–800	1.4–12	43–146.4	0.5–9	250–350	591–10,000	^<^ 60–3200	2–4	^<^ 427–15,000	1–3.5	80–100
	Paper Honeycomb	10–321	1–20	16.6–100	0.05–4.5	–	100–1680	^<^ 94.8–4200	^<^ 4.8–12	49.3–22,700	–	100

D: Density, AS: Average shrinkage, WP: Water absorption, VS: Volumetric swelling, TD: Thermal degradation, BS: Bending strength, CS: Compression strength, IS: Impact strength, TS: Tensile strength, EAB: Elongation at break, and B: Biodegradability. – is not reported, ^<^ is less than, and NB is non-biodegradable during 30 years. * [19,22,24,29,42,55,58,59,60,61,68,75,76,77,83,89,90,91,92,93,94,95,96,97,98,99,100,101,102,103,104,105,106,107,108,109,110,111,112,113,114,115,116,117,118,119,120,121,122,123].

## Data Availability

Data are contained within the article.

## References

[1] Rendón-Villalobos R., Ortíz-Sánchez A., Tovar-Sánchez E., Flores-Huicochea E., Matheus P. (2016). The role of biopolymers in obtaining environmentally friendly materials. Composites from Renewable and Sustainable Materials.

[2] Garcia-Garcia D., Quiles-Carrillo L., Balart R., Torres-Giner S., Arrieta M.P. (2022). Innovative solutions and challenges to increase the use of Poly (3-hydroxybutyrate) in food packaging and disposables. Eur. Polym. J..

[3] Zhang L., Xu M., Chen H., Li Y., Chen S. (2022). Globalization, green economy and environmental challenges: State of the art review for practical implications. Front. Environ. Sci..

[4] Mohamed F., Jamil M., Zain M.F.M. (2019). Sustainable material: Challenges and prospect. J. Adv. Res. Mater. Sci..

[5] Obeidat I., Obeidat S., Rumman S.A., Al-Jubouri F. The role of sustainable interior design and its impact on customer’s behavior in commercial environments. Proceedings of the IOP Conference Series: Earth and Environmental Science.

[6] Paulraj P., Ilangovan P., Subramanian K., Nagarajan M.R., Suthan R., Sakthimurugan V., Madhu S., Varuvel E.G., Lenin H. (2023). Environmentally conscious manufacturing and life cycle analysis: A state-of-the-art survey. J. Nanomater..

[7] Khalil H.A., Bhat A.H., Yusra A.I. (2012). Green composites from sustainable cellulose nanofibrils: A review. Carbohydr. Polym..

[8] Abdur Rahman M., Haque S., Athikesavan M.M., Kamaludeen M.B. (2023). A review of environmental friendly green composites: Production methods, current progresses, and challenges. Environ. Sci. Pollut. Res..

[9] Livne A., Wösten H.A., Pearlmutter D., Gal E. (2022). Fungal mycelium bio-composite acts as a CO_2_-sink building material with low embodied energy. ACS Sustain. Chem. Eng..

[10] Sydor M., Cofta G., Doczekalska B., Bonenberg A. (2022). Fungi in mycelium-based composites: Usage and recommendations. Materials.

[11] Manan S., Ullah M.W., Ul-Islam M., Atta O.M., Yang G. (2021). Synthesis and applications of fungal mycelium-based advanced functional materials. J. Bioresour. Bioprod..

[12] Rajendran R.C., Sunil K.D., Mukund V.D., Kandikere R.S. (2022). Packaging Applications of Fungal Mycelium-Based Biodegradable Composites. Fungal Biopolymers and Biocomposites: Prospects and Avenues.

[13] Alemu D., Tafesse M., Mondal A.K. (2020). Mycelium-based composite: The future sustainable biomaterial. Int. J. Biomater..

[14] Kundanati L. (2022). Fungi-based biomimetic approach to address plastic pollution: A developing nation’s perspective. Preprints.

[15] Attias N., Danai O., Tarazi E., Pereman I., Grobman Y.J. (2019). Implementing bio-design tools to develop mycelium-based products. Des. J..

[16] Aiduang W., Kumla J., Srinuanpan S., Thamjaree W., Lumyong S., Suwannarach N. (2022). Mechanical, physical, and chemical properties of mycelium-based composites produced from various lignocellulosic residues and fungal species. J. Fungi.

[17] Kupradi C., Khongla C., Musika S., Ranok A., Tamaruay K., Woraratphoka J., Mangkalanan S. (2017). Cultivation of Lentinus squarrosulus and Pleurotus ostreatus on cassava bagasse based substrates. Int. J. Agric. Technol..

[18] (2000). Evaluation of the Action of Microorganisms on Plastics.

[19] Juanga-Labayen J.P., Yuan Q. (2021). Making biodegradable seedling pots from textile and paper waste—Part B: Development and evaluation of seedling pots. Int. J. Environ. Res. Public Health.

[20] McGowan M.J., Shimoda L.M., Woolsey G.D. (1988). Effects of sodium hypochlorite on denture base metals during immersion for short-term sterilization. J. Prosthet. Dent..

[21] (1999). Standard Test Method for Moisture Content of Paper and Paperboard by Oven Drying.

[22] Zimele Z., Irbe I., Grinins J., Bikovens O., Verovkins A., Bajare D. (2020). Novel mycelium-based biocomposites (Mbb) as building materials. J. Renew. Mater..

[23] Elsacker E., Vandelook S., Brancart J., Peeters E., Laet L.D. (2019). Mechanical, physical and chemical characterisation of myce-lium–based composites with different types of lignocellulosic substrates. PLoS ONE..

[24] Appels F.V.W., Camere S., Montalti M., Karana E., Jansen K.M.B., Dijksterhuis J., Krijgsheld P., Wosten H.A.B. (2019). Fabrication factors influencing mechanical, moisture and water related properties of mycelium-based composites. Mater. Des..

[25] Sandak A., Sandak J., Modzelewska I. (2019). Manufacturing fit-for-purpose paper packaging containers with controlled biodegradation rate by optimizing addition of natural fillers. Cellulose.

[26] Yang L., Park D., Qin Z. (2021). Material function of mycelium-based bio-composite: A review. Front. Mater. Sci..

[27] Deacon J.W. (2006). Fungal Biology.

[28] Velasco P.M., Ortiz M.P.M., Giro M.A.M., Castelló M.C.J., Velasco L.M. (2014). Development of better insulation bricks by adding mushroom compost wastes. Energy Build..

[29] De Lima G.G., Schoenherr Z.C.P., Magalhães W.L.E., Tavares L.B.B., Helm C.V. (2020). Enzymatic activities and analysis of a mycelium-based composite formation using peach palm (*Bactris gasipaes*) residues on Lentinula edodes. Bioresour. Bioprocess.

[30] Aiduang W., Chanthaluck A., Kumla J., Jatuwong K., Srinuanpan S., Waroonkun T., Oranratmanee W., Lumyong S., Suwannarach N. (2022). Amazing fungi for eco-friendly composite materials: A comprehensive review. J. Fungi.

[31] Houette T., Maurer C., Niewiarowski R., Gruber P. (2022). Growth and mechanical characterization of mycelium-based composites towards future bioremediation and food production in the material manufacturing cycle. Biomimetics.

[32] Aiduang W., Suwannarach N., Kumla J., Thamjaree W., Lumyong S. (2022). Valorization of agricultural waste to produce myco-composite materials from mushroom mycelia and their physical properties. Agric. Nat. Resour..

[33] Holt G.A., Mcintyre G., Flagg D., Bayer E., Wanjura J.D., Pelletier M.G. (2012). Fungal mycelium and cotton plant materials in the manufacture of biodegradable molded packaging material: Evaluation study of select blends of cotton byproducts. J. Biobased Mater..

[34] Rigobello A., Ayres P. (2022). Compressive behaviour of anisotropic mycelium-based composites. Sci. Rep..

[35] Butu A., Rodino S., Miu B., Butu M. (2020). Mycelium-based materials for the ecodesign of bioeconomy. Dig. J. Nanomater. Biostructures.

[36] Gou L., Li S., Yin J., Li T., Liu X. (2021). Morphological and physico-mechanical properties of mycelium biocomposites with natural reinforcement particles. Constr. Build. Mater..

[37] Tacer-Caba Z., Varis J.J., Lankinen P., Mikkonen K.S. (2020). Comparison of novel fungal mycelia strains and sustainable growth substrates to produce humidity-resistant biocomposites. Mater. Des..

[38] Joshi K., Meher M.K., Poluri K.M. (2020). Fabrication and characterization of bioblocks from agricultural waste using fungal mycelium for renewable and sustainable applications. ACS Appl. Bio Mater..

[39] Angelova G., Brazkova M., Stefanova P., Blazheva D., Vladev V., Petkova N., Slavov A., Denev P., Karashanova D., Zaharieva R. (2021). Waste rose flower and lavender straw biomass—An innovative lignocellulose feedstock for mycelium bio-materials development using newly isolated Ganoderma resinaceum GA1M. J. Fungi.

[40] Chan X.Y., Saeidi N., Javadian A., Hebel D.E., Gupta M. (2021). Mechanical properties of dense mycelium-bound composites under accelerated tropical weathering conditions. Sci. Rep..

[41] Jones M., Mautner A., Luenco S., Bismarck A., John S. (2020). Engineered mycelium composite construction materials from fungal biorefineries: A critical review. Mater. Des..

[42] Pishan S., Ghofrani M., Kermanian H. (2014). Study on mechanical properties of lightweight panels made of honeycomb and polyurethane cores. Lignocellulose.

[43] Paperonweb Typical Density and Bulk of Paper. https://www.paperonweb.com/density.htm#a.

[44] Alaneme K.K., Anaele J.U., Oke T.M., Kareem S.A., Adediran M., Ajibuwa O.A., Anabaranze Y.O. (2023). Mycelium based composites: A review of their bio-fabrication procedures, material properties and potential for green building and construction applications. Alex. Eng. J..

[45] Kim D. (2018). Physico-chemical conversion of lignocellulose: Inhibitor effects and detoxification strategies: A mini review. Molecules.

[46] Ibrahim M.I., Sapuan S.M., Zainudin E.S., Zuhri M.Y.M. (2019). Extraction, chemical composition, and characterization of potential lignocellulosic biomasses and polymers from corn plant parts. Bioresources.

[47] Zhai Q., Long F., Hse C.Y., Wang F., Shupe T.F., Jiang J., Xu J. (2019). Facile fractionation of bamboo wood toward biomass valorization by p-TsOH-based methanolysis pretreatment. ACS Sustain. Chem. Eng..

[48] Zhang K., Li H., Xiao L.P., Wang B., Sun R.C., Song G. (2019). Sequential utilization of bamboo biomass through reductive catalytic fractionation of lignin. Bioresour. Technol..

[49] Pantamanatsopa P., Ariyawiriyanan W., Sungsanit K., Ekgasit S. (2023). Physicochemical characterization of acid-treated nanocrystal cellulose and amorphous cellulose from bamboo sawdust. J. Nat. Fibers.

[50] Peeters S.S. (2023). Assessing Modifications on Mycelium-Based Composites and the Effects on Fungal Degradation and Material Properties. Master’s Thesis.

[51] Zhang X., Fan X., Han C., Wang C., Yu X. Improving soil surface erosion resistance by fungal mycelium. Proceedings of the Geo-Congress 2020, VA: American Society of Civil Engineers.

[52] Robertson O., Høgdal F., Mckay L., Lenau T. Fungal Future: A review of mycelium biocomposites as an ecological alter-native insulation material. Proceedings of the Nord Design 2020.

[53] Sun W., Tajvidi M., Hunt C.G., McIntyre G., Gardner D.J. (2019). Fully bio-based hybrid composites made of wood, fungal mycelium and cellulose nanofibrils. Sci. Rep..

[54] Sun W. (2021). Understanding the Adhesion Mechanism in Mycelium-Assisted Wood Bonding. Ph.D. Thesis.

[55] Charpentier-Alfaro C., Benavides-Hernández J., Poggerini M., Crisci A., Mele G., Della Rocca G., Emiliani G., Frascella A., Torrigiani T., Palanti S. (2023). Wood-decaying fungi: From timber degradation to sustainable insulating biomaterials production. Materials.

[56] Bruscato C., Malvessi E., Brandalise R.N., Camassola M. (2019). High performance of macrofungi in the production of mycelium-based biofoams using sawdust—Sustainable technology for waste reduction. J. Clean. Prod..

[57] Waters C.L., Janupala R.R., Mallinson R.G., Lobban L.L. (2017). Staged thermal fractionation for segregation of lignin and cellulose pyrolysis products: An experimental study of residence time and temperature effects. J. Anal. Appl. Pyrolysis.

[58] Agarwal G.A.U.R.A.V., Liu G., Lattimer B.R.I.A.N. (2014). Pyrolysis and oxidation of cardboard. Fire Saf. Sci..

[59] Chen N., Zhang S., Pan X., Zhou S., Zhao M. (2020). Foaming mechanism and optimal process conditions of foamed glass based on thermal analysis. J. Porous Mater..

[60] Ma Y., Song D., Cao J. (2020). Preparation of activated carbon monolith from waste corrugated cardboard box via catalytic pyrolysis and gasification under CO_2_ atmosphere for adsorption and solar steam generation. J. Porous Mater..

[61] Nazir M.T., Phung B.T., Yeoh G.H., Yasin G., Akram S., Bhutta M.S., Mehmood M.A., Hussain S., Yu S., Kabir I. (2020). Enhanced dielectric and thermal performance by fabricating coalesced network of alumina trihydrate/boron nitride in silicone rubber for electrical insulation. Bull. Mater. Sci..

[62] Rasid Z.A. (2015). The Thermal Stability Property of Bio-composites: A Review. InCIEC 2014: Proceedings of the International Civil and Infrastructure Engineering Conference 2014.

[63] Sun W., Tajvidi M., Howell C., Hunt C.G. (2022). Insight into mycelium-lignocellulosic bio-composites: Essential factors and properties. Compos. A Appl. Sci. Manuf..

[64] Raimbault M. (2003). General and microbiological aspects of solid substrate fermentation. Electron. J. Biotechnol..

[65] Wösten H.A. (2019). Filamentous fungi for the production of enzymes, chemicals and materials. Curr. Opin. Biotechnol..

[66] Elsacker E., Vandelook S., Damsin B., Van Wylick A., Peeters E., De Laet L. (2021). Mechanical characteristics of bacterial cellulose-reinforced mycelium composite materials. Fungal Biol. Biotechnol..

[67] Akromah S., Chandarana N., Eichhorn S.J. (2023). Mycelium composites for sustainable development in developing countries: The case for Africa. Adv. Sustain. Syst..

[68] MatWeb LLC Material Property Data. https://www.matweb.com/search/AdvancedSearch.aspx.

[69] Javadian A., Le Ferrand H., Hebel D.E., Saeidi N. (2020). Application of mycelium-bound composite materials in construction industry: A short review. SOJ Mater. Sci. Eng..

[70] Pohl C., Schmidt B., Nunez Guitar T., Klemm S., Gusovius H.J., Platzk S., Kruggel-Emden H., Klunker A., Völlmecke C., Fleck C. (2022). Establishment of the basidiomycete Fomes fomentarius for the production of composite materials. Fungal Biol. Biotechnol..

[71] Kohphaisansombat C., Jongpipitaporn Y., Laoratanakul P., Tantipaibulvut S., Euanorasetr J., Rungjindamai N., Chuaseeharonnachaid C., Kwantong P., Somrithipol S., Boonyuen N. (2023). Fabrication of mycelium (oyster mushroom)-based composites derived from spent coffee grounds with pineapple fibre reinforcement. Mycology.

[72] Vašatko H., Gosch L., Jauk J., Stavric M. (2022). Basic research of material properties of mycelium-based composites. Biomimetics.

[73] Sivakumar D., Kathiravan S., Ng L.F., Ali M.B., Selamat M.Z., Sivaraos S., Bapokutty O. (2018). Experimental investigation on charpy impact response of kenaf bast fibre reinforced metal laminate system. ARPN J. Eng. Appl. Sci..

[74] Abidin N.M.Z., Sultan M.T.H., Shah A.U.M., Safri S.N.A. Charpy and Izod impact properties of natural fibre composites. Proceedings of the 6th International Conference on Applications and Design in Mechanical Engineering.

[75] Barboutis I., Vassiliou V. Strength properties of lightweight paper honeycomb panels for the furniture. Proceedings of the International Scientific Conference.

[76] Ashby M.F. (2012). Materials and the Environment: Eco-Informed Material Choice.

[77] Keskisaari A., Kärki T., Vuorinen T. (2019). Mechanical properties of recycled polymer composites made from side-stream materials from different industries. Sustainability.

[78] Baca Lopez D.M., Ahmad R. (2020). Tensile mechanical behaviour of multi-polymer sandwich structures via fused deposition modelling. Polymers.

[79] Gauvin F., Vette I.J. (2020). Characterization of Mycelium-Based Composites as Foam-Like Wall Insulation Material. Master’s Thesis.

[80] Travaglini S., Noble J., Ross P.G., Dharan C.K.H. Mycology matrix composites. Proceedings of the Annual Technical Conference 28th American Society for Composites.

[81] Kopal I., Labaj I., Harničárová M., Valíček J., Hrubý D. (2018). Prediction of the tensile response of carbon black filled rubber blends by artificial neural network. Polymers.

[82] Czechowski L., Śmiechowicz W., Kmita-Fudalej G., Szewczyk W. (2020). Flexural damage of honeycomb paperboard—A numerical and experimental study. Materials.

[83] Pohl A. (2009). Strengthened Corrugated Paper Honeycomb for Application in Structural Elements. Ph.D. Thesis.

[84] Sisti L., Gioia C., Totaro G., Verstichel S., Cartabia M., Camere S., Celli A. (2021). Valorization of wheat bran agro-industrial byproduct as an upgrading filler for mycelium-based composite materials. Ind. Crops Prod..

[85] Van Wylick A., Elsacker E., Yap L.L., Peeters E., De Laet L. (2022). Mycelium composites and their biodegradability: An exploration on the disintegration of mycelium-based materials in soil. Constr. Technol. Archit..

[86] Ly L., Jitjak W. (2022). Biocomposites from agricultural wastes and mycelia of a local mushroom, *Lentinus squarrosulus* (Mont.) Singer. Open Agric..

[87] Kjeldsen A., Price M., Lilley C., Guzniczak E., Archer I. (2018). A review of standards for biodegradable plastics. Ind. Biotechnol. Innov. Cent..

[88] Fairus M.J.B.M., Bahrin E.K., Arbaain E.N.N., Ramli N.O.R.H.A.Y.A.T.I., Enis N. (2022). Mycelium-based composite: A way forward for renewable material. J. Sustain. Sci. Manag..

[89] Bian J., Cao W., Yang L., Xiong C. (2018). Experimental research on the mechanical properties of tailing microcrystalline foam glass. Material.

[90] Armstrong S. (2016). Foamglas Cellular Glass Insulation Is Proven Value.

[91] Şahin A., Kılıç Y., Kara M., Sarı A., Duymaz B. An investigation of raw material effects on nano sic based foam glass production. Proceedings of the Conference: SERES’18 IV International Ceramic, Glass, Porcelain Enamel, Glaze and Pigment Congress.

[92] Scarinci G., Brusatin D., Bernardo E., Michael S., Paolo C. (2005). Glass Foams. Cellular Ceramics: Structure, Manufacturing, Properties and Applications.

[93] Yang L., Gao J., Liu Y., Zhuang G., Peng X., Wu W.M., Zhuang X. (2021). Biodegradation of expanded polystyrene and low-density polyethylene foams in larvae of *Tenebrio molitor* Linnaeus (Coleoptera: Tenebrionidae): Broad versus limited extent depolymerization and microbe-dependence versus independence. Chemosphere.

[94] Maghfouri M., Alimohammadi V., Gupta R., Saberian M., Azarsa P., Hashemi M., Asadi I., Roychand R. (2022). Drying shrinkage properties of expanded polystyrene (EPS) lightweight aggregate concrete: A review. Case Stud. Constr. Mater..

[95] Zegardło B., Kobyliński K. (2021). Analysis of the possibility of using extruded polystyrene (xps) wastes to make lightweight cement composites. J. Ecol. Eng..

[96] Plascams Phenolic PF Phenol Formaldehyde Foam. https://www.azom.com/article.aspx?ArticleID=728.

[97] Xu Y., Guo L., Zhang H., Zhai H., Ren H. (2019). Research status, industrial application demand and prospects of phenolic resin. RSC Adv..

[98] Lampé I., Hegedús C. (2002). Comparative evaluation of the shrinkage of addition-type silicone impression material using hand-mix and cartridge-mix technique. Fogorv. Sz..

[99] Russ A., Schwartz J., Boháček Š., Lübke H., Ihnat V., Pažitný A. (2013). Reuse of old corrugated cardboard in constructional and thermal insulating boards. Wood Res..

[100] Łątka J.F., Jasiołek A., Karolak A., Niewiadomski P., Noszczyk P., Klimek A., Zielińska S., Misiurka S., Jezierska D. (2022). Properties of paper-based products as a building material in architecture–An interdisciplinary review. J. Build. Eng..

[101] Elliott O. (2022). The Benefits of Cardboard Packaging. https://www.bigdug.co.uk/blog/benefits-of-cardboard-packaging/.

[102] Mahakalkar S.G., Sambare R., Sunheriya N. (2019). Effect of environmental conditions on performance of corrugated sheet boxes manufacturing process. Int. J. Recent. Technol. Eng..

[103] Tungsangprateep S., Kulchan R., Toonkham W., Tuntawiroon O., Meaktrong W., Praditniyakul B., Maneesin P., Sansupa S. (2006). Development of consumer packaging for fresh persimmons. Agric. Nat. Resour..

[104] Wendler S. (2006). Washboarding of Corrugated Cardboard. Ph.D. Thesis.

[105] Ralph A. Swelling of Corrugated Boxes. https://askralph-aiccbox.org/2015/03/02/swelling-of-corrugated-boxes/.

[106] Fadiji T., Berry T., Coetzee C.J., Opara L. (2017). Investigating the mechanical properties of paperboard packaging material for handling fresh produce under different environmental conditions: Experimental analysis and finite element modelling. J. Appl. Packag. Res..

[107] Zulaikah S., Triawan F., Budiman B.A., Romadhon Y., Kamaludin D., Juraj M., Gregor P., Ulrike G., Faisal M. (2023). Study on the mechanical properties and behavior of corrugated cardboard under tensile and compression loads. Materials Science Forum.

[108] Allaoui S., Aboura Z., Benzeggagh M.L. (2009). Phenomena governing uni-axial tensile behaviour of paperboard and corrugated cardboard. Compos. Struct..

[109] Korte I., Albrecht A., Mittler M., Waldhans C., Kreyenschmidt J. (2023). Influence of different bio-based and conventional packaging trays on the quality loss of fresh cherry tomatoes during distribution and storage. Packag Technol. Sci..

[110] Fefco (2020). The Study Biodegradability and Compostability Fefco. https://www.gifco.it/wp-content/uploads/2020/11/Fefco_ppt_report_biodegradability_and_compostability_2020.pdf.

[111] Odusote J.K., Onowuma S.A., Fodeke E.A. (2016). Production of paperboard briquette using waste paper and sawdust. J. Eng. Res..

[112] Larsson P.A. (2008). Dimensional Stability of Paper: Influence of Fibre-Fibre Joints and Fibre Wall Oxidation. Ph.D. Thesis.

[113] Odusote J.K., Dosunmu K.S. (2019). Development of chicken feather reinforced insulation paperboard from waste carton and Portland cement. J. Eng. Res..

[114] Ataguba C.O. (2016). Properties of ceiling boards produced from a composite of waste paper and rice husk. Int. J. Adv. Sci. Eng. Technol..

[115] Marin G., Nygårds M., Östlund S. (2022). Experimental quantification of differences in damage due to in-plane tensile test and bending of paperboard. Packag. Technol. Sci..

[116] Naitzel T.D.C., Garcia V.A.D.S., Lourenço C.A.M., Vanin F.M., Yoshida C.M.P., Carvalho R.A.D. (2023). Properties of paperboard coated with natural polymers and polymer blends: Effect of the number of coating layers. Foods.

[117] Rhim J.W., Lee J.H., Hong S.I. (2007). Increase in water resistance of paperboard by coating with poly (lactide). Packag. Technol. Sci..

[118] Sridach W., Retulainen E., Nazhad M.M., Kuusipalo J., Parkpian P. (2006). Biodegradable barrier coating on paperboard: Effects on biodegradation, recycling and incineration. Pap. Ja Puu.

[119] Semple K.E., Sam-Brew S., Deng J., Cote F., Yan N., Chen Z., Smith G.D. (2015). Properties of commercial kraft paper honeycomb furniture stock panels conditioned under 65 and 95 percent relative humidity. For. Prod. J..

[120] Ayrilmis N., Kuzman M.K. (2016). Properties of honeycomb paperboards faced with heat-treated thin medium-density fiberboards. Bioresources.

[121] Fu Y., Sadeghian P. (2023). Bio-based sandwich beams made of paper honeycomb cores and flax FRP facings: Flexural and shear characteristics. Structures.

[122] Słonina M., Dziurka D., Molińska-Glura M., Smardzewski J. (2022). Influence of impregnation with modified starch of a paper core on bending of wood-based honeycomb panels in changing climatic conditions. Materials.

[123] Bewi (2023). Honeycomb. https://bewi.com/material/honeycomb/.

[124] Bitting S., Derme T., Lee J., Van Mele T., Dillenburger B., Block P. (2022). Challenges and opportunities in scaling up architectural applications of mycelium-based materials with digital fabrication. Biomimetics.

[125] Protopapadaki I., Kalika S. Insights into Mycelium. https://criticalconcrete.com/insights-mycelium/.

[126] Bonenberg A., Sydor M., Cofta G., Doczekalska B., Grygorowicz-Kosakowska K. (2023). Mycelium-based composite materials: Study of acceptance. Materials.

[127] Teeraphantuvat T., Jatuwong K., Jinanukul P., Thamjaree W., Lumyong S., Aiduang W. (2024). Improving the physical and mechanical properties of mycelium-based green composites using paper waste. Polymers.

